# Experiences of women with gestational diabetes about fear of having diabetes in their babies: A qualitative study

**DOI:** 10.1097/MD.0000000000037755

**Published:** 2024-04-12

**Authors:** Ekin Dila Topaloğlu Ören, Elif Ünsal Avdal, Gökşen Polat, Funda Sofulu, Gönül Düzgün, Gülseren Pamuk

**Affiliations:** a Izmir Katip Celebi University Faculty of Health Science, Department of Obstetrics and Gynecology Nursing, İzmir, Türkiye; b Izmir Katip Celebi University Faculty of Health Science, Department of Internal Medicine Nursing, İzmir, Türkiye; c Izmir Tinaztepe University Faculty of Health Science, Department of Internal Medicine Nursing, İzmir, Türkiye; d İzmir Tinaztepe University Faculty of Health Science, Department of First Aid and Emergency, İzmir, Türkiye; e Izmir Katip Celebi University School of Medicine, Department of Family Medicine, İzmir, Türkiye.

**Keywords:** fear, gestational diabetes, phenomenology, pregnancy, pregnant women, Türkiye

## Abstract

Gestational Diabetes Mellitus is an important public health problem that often occurs during pregnancy. This study aimed to reveal the experiences of women with gestational diabetes regarding the fear of having diabetes in their babies. A qualitative research method was carried out with a phenomenological approach. The interviews had a semi-structured form and were recorded on an online/face-to-face voice recorder, and thematic content analysis was performed on the MAXQDA22. Following the inclusion criteria, 12 women with gestational diabetes from the 2 hospitals in the study were included, and in this way, the study reached saturation. As a result of the interviews, 4 main themes and one subtheme were obtained from coding. The main themes were “sugar baby,” “risky child,” “raising a fearful baby,” and “problematic gene carrier.” From the main theme of “problematic gene carrier,” the subtheme of “pregnancies with problematic genes” was created. This research sheds light on the problems women with gestational diabetes experience with themselves and their babies, and how they deal with these problems. Women with gestational diabetes try to accept and cope with the diagnosis. This research shows that the women were worried about both themselves and their babies. Illuminating the experiences of women with gestational diabetes is part of an integrative care approach that will help increase quality care and treatment in endocrine clinics. More qualitative studies are needed to learn more about the experiences of women with gestational diabetes in endocrine clinics.

## 1. Introduction

Diabetes is one of the most common systemic diseases in pregnancy.^[1]^ Gestational Diabetes Mellitus (GDM) is carbohydrate intolerance that first appears during pregnancy and usually disappears spontaneously with the birth of the baby.^[2,3]^ GDM is a form of diabetes associated with varying degrees of beta cell dysfunction in clinical presentation and progression and usually occurs in the second trimester of pregnancy. GDM is associated with pregnancy-related insulin resistance and genetic predisposition.^[1,4]^

In the physiopathology of GDM, the use of glucose in the periphery increases in the first trimester of pregnancy and causes a decrease in fasting blood sugar levels. This decline begins at the eighth week of pregnancy and continues until the 12th week.^[4,5]^ On the other hand, a slow rise in postprandial blood glucose level is observed due to delayed gastric emptying. Basal glucose level and insulin concentrations are the same as before pregnancy.^[6]^ This is because estrogen and progesterone increase pancreatic beta-cell hyperplasia. This increase in pancreatic beta cell mass leads to increased insulin secretion.^[5,7]^ In the third trimester, maternal insulin resistance increases with the anti-insulin effects of estrogen, cortisol, prolactin, corticotropin, progesterone, and especially human placental lactogen.^[8,9]^ Increased insulin resistance and increased hepatic glucose production in the liver cause an increase in fasting blood glucose levels. This leads to a decrease in glucose uptake by the muscle layer and, accordingly, to an increase in postprandial glucose levels.^[10–12]^ Most of the diabetes encountered during pregnancy is GDM (90%), while 10% of GDM is pregestational diabetes mellitus.^[13–15]^ In studies conducted in Türkiye, the prevalence of GDM was 16% to 24.8%.^[16–18]^ The risk factors for GDM were obesity,^[16,19,20]^ sedentary life,^[16,19]^ age at menarche < 12 years,^[20]^ number of pregnancies of 4 or more,^[16]^ history of macrosomic infant birth,^[19]^ family history of diabetes/ethnicity,^[16,19]^ and high fasting blood glucose.^[16,19]^ In addition, the prevalence of GDM was significantly higher in pregnant women with a history of stillbirth or neonatal death and birth defects in previous births.^[16]^

GDM is defined as glucose intolerance of varying degrees that first appears during pregnancy.^[6]^ The incidence of GDM varies from society to society, but its incidence is increasing. The most important reason for this is the increasing incidence of obesity worldwide and the decrease in threshold values in diagnostic tests.^[21,22]^ The American Diabetes Association reports that GDM is detected in approximately 4% of pregnant women, or 135,000 women per year.^[23]^ The incidence of GDM differs according to race,^[24]^ and it increases with age. Women over the age of 25 are at greater risk of GDM than women under 25.^[21,23]^

It was reported in a study that GDM is more common in women between the ages of 25 and 45 who have a body mass index of 40 and above.^[25,26]^ GDM is an important health problem because it is frequently seen and affects mother-infant health. Women with GDM are at higher risk of maternal and neonatal complications than those without GDM. GDM increases the risk of preeclampsia,^[27]^ cesarean delivery,^[1,27]^ type 2 diabetes,^[28]^ and prenatal and postpartum depression in pregnant women.^[29]^ In infants, it can cause macrosomia,^[1,27,28]^ prematurity,^[1,27,28]^ respiratory distress syndrome,^[1,27]^ hiperbilürinemi,^[1,27]^ 5 minutes Apgar < 7,^[27]^ neonatal hypoglycemia,^[27]^ hyperglycemia,^[28]^ shoulder dystocia,^[27]^ childhood obesity,^[1]^ prediabetes, type 2 diabetes,^[28]^ and increased the risk of hospitalization in the neonatal care unit.^[1,27,28]^ One of the important problems experienced by women with GDM is the fear of their babies becoming diabetic. Women with GDM may experience fear during pregnancy that their baby will be born with diabetes and that they will harm their baby.^[13,30–33]^ Carson et al reported that women with GDM experienced fear for the health of their babies and described diabetes as a severe disease.^[34]^ Hirst et al reported in a qualitative study that pregnant women felt confusion, anxiety, and guilt about GDM, feared for the health of their babies, worried that diabetes would pass to the baby through breast milk, and did not want to breastfeed.^[35]^ Lapolla et al reported that one-third of women with GDM experienced diabetes and fear of congenital malformations in their babies.^[36]^ Nolan et al found that women with GDM were afraid that diabetes might adversely affect the health of their babies before, during, and after birth and in the following years.^[37]^ Women with GDM experience anxiety and fear about their babies for different reasons. One of these is the fear that their babies will have diabetes. This fear experienced by women with GDM negatively affects both their health and the health of their babies. To improve these results, healthcare providers, especially nurses, should regularly monitor women with GDM during pregnancy and the postpartum period, inform them about the effects of GDM on mother and baby, and develop strategies to cope with GDM. Nurses should be aware of the fears of women with GDM about themselves and their babies, support them, and provide education and counseling.^[13,25,38]^ The life experiences of women with GDM in Türkiye regarding the fear of their babies having diabetes have not been studied before. This study revealed the thoughts, feelings, and expectations about diabetes of women with GDM. Thus, the study aimed to examine with a phenomenological approach the fear of women with GDM of having children with diabetes. The study aimed to examine the fear of their children having diabetes in women with gestational diabetes with a phenomenological approach.

## 2. Materials and methods

### 2.1. Study design

This research was carried out in the phenomenology design, which is one of the qualitative research methods. Phenomenological research focuses on what the participants’ experiences are, how they describe their experiences, and how these experiences affect them. Phenomenological research specifically examines the participants’ perspectives on events and the meaning of events for them.^[39]^ The descriptive phenomenological study designs offer comprehensive information about an event. Also, it is recommended for researchers intending to investigate situation-specific factors and to evaluate their impact on people.^[40]^ Thus, the nature of this study is based on understanding the core experiences, thoughts, and feelings of women with GDM about the fear of having children with diabetes. In the reporting of this study, the Consolidated Criteria for Reporting Qualitative Research was used as a guideline.^[41]^

### 2.2. Study sample

This study was conducted in the endocrine clinics of a private hospital Izmir, western Türkiye, between January 2023 and February 2023 with 12 women with gestational diabetes. The purposeful sampling method was used to identify a sample that met the predetermined and important criteria. The sample criteria were being diagnosed with gestational diabetes for the first time, being between the ages of 18 to 35, having a live birth after diagnosis, having given birth a maximum of 2 years ago, and volunteering to take part in the study. Exclusion criteria were having been previously diagnosed with gestational diabetes, having a chronic disease other than gestational diabetes, being under the age of 18 or over the age of 35, and having given birth more than 2 years previously. As a result of the in-depth interview method with women with GDM in the research, new information ceased to be obtained, the information began to be repeated, and the saturation point was reached with 12 women. The purposeful sampling method was used to reach this number.

### 2.3. Data collection

Data were collected by in-depth interview method using semi-structured questions and observation notes in January 2023 and February 2023 in Izmir in the western of Türkiye. The data of the research were collected with the Individual Introduction Form and the Semi-Structured Interview Form.

The Individual Introduction Form was prepared by the researchers based on previous studies. The form consists of 6 questions about *age, education, working, number of pregnancies, number of living children*, and *family history of diabetes*. The Semi-Structured Interview Form consists of 8 open-ended semi-structured questions concerning the experiences, expectations, feelings, and thoughts about the fear of diabetes in the children of women with GDM. The Semi-Structured Interview Form was prepared by the researchers based on previous studies.^[10,25,35,42,43]^ and evaluated for content and scope by an expert. In addition, a pilot study (preinterview) was conducted before starting the research with 2 women with GDM. The interview form was given its final form as a result of expert opinions and preliminary research. The Semi-Structured Interview Form includes 8 questions created to determine the experiences of women with GDM. The interviews lasted an average of 45 minutes and were conducted by the first researcher. The researchers observed each participant during the interviews, took observation notes, and audio-recorded the interviews. The names of the participants in the study were kept confidential. The statements of the 12 women with GDM were given by coding as P1, P2.

Data were collected in the research through the in-depth interview method using a semi-structured interview form with 12 women with GDM. Before the interviews, the women who had given birth, been diagnosed with GDM, and met the inclusion criteria of the study were determined, the purpose of the research was explained over the phone and it was determined whether they were willing to participate. Individuals who agreed to participate in the study were interviewed. A single researcher to increase reliability and consistency collected the interviews. Interviews were conducted at a time convenient to the participant and in a quiet environment, with audio recordings. The data collection process continued until the saturation level was reached in the data.

#### 2.3.1. Interview questions

1. *What do you know about gestational diabetes?*
2. *How did gestational diabetes affect your pregnancy?*
3. *As a mother with gestational diabetes, do you think your baby may have diabetes?*
4. *After the diagnosis of gestational diabetes, do you take precautions to prevent your baby from having diabetes?*
5. *What are the reasons for your fear and anxiety that your baby has diabetes?*
6. *A mother with gestational diabetes, do you think anything will happen to your baby in your next birth?*
7. *Who do you get help from if your baby has diabetes?*
8. *How do you think being diagnosed with gestational diabetes affects your baby’s health?*

### 2.4. Data analysis

In the analysis of the data, after the audio recordings of the interviews were deciphered, thematic content analysis was carried out in MAXQDA 22, a qualitative data analysis program, and then the themes and subthemes that would represent these codes were determined. The researchers read the transcripts many times and inductive coding was performed independently. From the beginning to the end of the research, all transcripts were reviewed by an academician in the field (co-supervisor). This was done to reveal and minimize the prejudices, socio-cultural perceptions, and assumptions of the researchers. The bracketing interviews created negotiated, supportive relationships that served as an interface between the researchers and the research data. In addition, the researchers wrote memos as a method of bracketing to means of examining and reflecting upon the researcher’s engagement throughout data collection and analysis. The transcripts were coded line by line by the researchers and the resulting codes were discussed face-to-face on subthemes and themes. The statements given by the participants were compared in terms of similarities and differences. After the forming of the final themes, they were translated from Turkish to English by a bilingual and native English speaker from outside the research team and reviewed by the research team.

### 2.5. Trustworthiness

In this research, the trustworthiness of the data was ensured in line with the principles of credibility, reliability, confirmability, transferability, and transparency.^[19–21]^ To increase the credibility of the research, a semi-structured interview form was developed in line with the literature, and a conceptual framework was created. More than one data collection method (individual interview and observation notes) was used in this study. To ensure its reliability, the participants were included in the study voluntarily and were asked to detail their experience in the process as much as possible. During the interviews, the researchers listened and observed objectively, without affirming or rejecting the participants’ experiences, thoughts, and feelings. Thus, the participants freely expressed their experiences. After each interview was completed, the interviews, which were transcribed verbatim, were sent back to the participants, and they were asked to check the accuracy and comprehensibility of the statements. After obtaining approval of the accuracy and comprehensibility of the statements, the data were analyzed (peer confirmation). Apart from the research, all transcripts were reviewed by an expert academician working in the same field (co-supervisor). Additionally, data source triangulation and researcher triangulation were used in the study. To increase the reliability of the research, the researchers carried out the thematic analysis process independently from each other. Afterward, the researchers held face-to-face and online meetings and repeatedly discussed the relationship between the identified themes, observation notes, and findings. Researchers had received training on conducting qualitative research, were experienced in qualitative research, had written a doctoral thesis, and had been working for many years as science specialists in the field of gynecology and obstetrics and internal diseases nursing and doctors.

### 2.6. Ethical considerations

Written permission was obtained from Izmir Kâtip Çelebi University Non-Interventional Clinical Research Ethics Committee and the hospital where the study was to be conducted (IRB: 0503/24/11/2022). The women who agreed to participate in the study were informed about the study and their written consent was obtained. The audio recordings of the interviews were only listened to by the researchers, the interviews were deciphered anonymously and the confidentiality of the information was ensured. Participants were informed that they could leave at any stage of the research. This study was carried out by the Declaration of Helsinki.

## 3. Results

The ages of the women with GDM in the study ranged from 23 to 35 years (N = 12). Eight of the women with GDM were graduates, 2 of them were undergraduates, and 2 of them were primary school graduates. All of the women were working; 7 of them had one living child and 5 had 2 living children. Only 5 of the women had a family history of diabetes. The mean number of pregnancies of the women was 2.48 ± 2.22.

Twelve women with GDM were included in the study. As a result of the analysis, 4 main themes were created with codes. These were “sugar baby,” “risky child,” “raising a fearful baby,” and “problem gene carrier.” From the main theme of “problematic gene carrier,” the subtheme of “pregnancies with problematic genes” was created.

### 3.1. Main theme: sugar baby

Women with GDM joked about their babies by using nicknames/labels as a way of coping with the diagnosis or to prevent the current situation from causing more stress on them.

‘Because it didn’t feel right to define it as a disease in this process, we said postpartum sugar baby’ (P1, 35), ‘ I know she has a sugar baby because it’s a diabetes pregnancy ‘ (P3, 30), ‘When my blood sugar rose, I was very afraid that her baby’s sugar would rise. They made me laugh because we would have a baby like sugar’ (P10, 25).

### 3.2. Main theme: child at risk

Women with GDM were more afraid of their babies’ health status than their anxiety. In particular, the words risky pregnancy and risky child/baby were used by all mothers.

‘There can be a lot of hearsay. We learned that some are right and some are wrong. They said I would have a fat baby. I’m raising my child with fear’ (P2, 33), ‘I knew it was a risky baby. I was afraid of premature birth’ (P4, 29), ‘It caused stress in my pregnancy. I got a little depressed. Just because my child would be at risk’ (P6, 23), ‘It being a risky pregnancy made me feel like I was raising a risky child’ (P12, 24).

### 3.3. Main theme: raising a fearful baby

The anxieties and worries of the women with GDM were not over postpartum, and they continued to fear that there would be a problem, especially with their baby’s growth and development, or that their children would be diagnosed with diabetes because of them.

‘Every time I go to the examination with my child I am afraid, I ask if there is any finding’ (P3, 30), ‘I am afraid that my child will have diabetes, both my father and my mother have it’ (P6, 23), ‘Now I check blood sugar now and then because I have diabetes’ (P7, 35), ‘Of course, a risky pregnancy means a risky baby. He may be diabetic or have other diseases’ (P8, 31), ‘I had difficulties while raising my child. I thought that he would be a risky child, I thought that his organs would be damaged’ (P9, 27), ‘I am still afraid that he will be a baby with diabetes risk. I had to start feeding early. I heard some information on the news that it may increase diabetes in the future (P12, 24).

### 3.4. Main theme: problem gene carrier

The women thought that the GDM was genetic: they thought that they were carrying a problematic gene and that it was familial. It is thought that they may have interpreted the risk of developing diabetes in their children by associating it with genetics.

‘If it is in our genes, this problem may also be in my child’ (P2, 33), ‘I think he will be diagnosed with diabetes. Because it’s family. I have this fear, that the problem may be caused by me because diabetes occurs during pregnancy (P3, 30), ‘It may be genetic. Now they say genetics in everything’ (P6, 23), ‘I was afraid that I was carrying a problematic gene. I am worried that it will also happen in my next pregnancies’ (P9, 27).

Pregnant women are afraid of being diagnosed with GDM in their next pregnancies, as the problematic gene causes the same thing in their next pregnancies. In this way, the subtheme of **“Pregnancy with Problem Genes”** was created under this main theme.

#### 3.4.1. Subtheme: problem gene-problem pregnancies

Pregnant women who are diagnosed with GDM have fear and anxiety or think that their next pregnancies will also receive the same diagnosis in the postpartum period.

‘I have a fear that it will also happen in my future pregnancies’ (P12, 24), ‘.I am worried that it will also happen in my next pregnancies..’ (P9, 27), ‘..I will be more careful in my next pregnancies..’ (P8, 31), ‘..it won’t happen in another pregnancy, right?..’ (P7, 35), ‘..it will be certain in my other pregnancies..’ (P6, 23), ‘..every pregnancy is different, but of course, it increases the risk..’ (P5, 32), ‘..I will have other pregnancies, I had my previous pregnancy too..’(P4, 29), ‘..I think that in my next pregnancies, I will also have diabetes again..’ (P3, 30).

## 4. Discussion

This was the first study in Türkiye to reveal the experiences, thoughts, and feelings about diabetes in the children of women with GDM. Women with GDM had some fears about their children having diabetes. Four main themes and one subtheme were obtained from coding in the study. The main themes were “sugar baby,” “risky child,” “raising a fearful baby,” and “problematic gene carrier.” From the main theme of “problematic gene carrier,” the subtheme of “pregnancies with problematic genes” was created.

In this study, the nature of these results was discussed. GDM is a common disease in Türkiye and worldwide, which, if not controlled, affects the health of both the pregnant woman and the fetus.^[13,14,21,23]^ When women with GDM first receive this diagnosis, they may experience many problems such as negative thoughts, difficulty in following the disease, stress, and anxiety. However, women with GDM were struggling to overcome these problems and to maintain a healthy pregnancy.^[10,42,43]^ In our study, it was concluded that pregnant women joke about their babies by using nicknames/labels to cope with the diagnosis or to prevent the current situation from stressing them more. In our study, the fact that pregnant women referred to their babies as “sugar babies” was a result of this. Women with GDM may have chosen this path to manage the process, normalize the disease, cope with the disease, reduce stress, and develop positive attitudes about the health of their babies.

GDM is one of the important health problems of women during pregnancy.^[21,23,44]^ The diagnosis of GDM was very difficult for a woman who planned to live her pregnancy uneventfully and peacefully, and it created anxiety and fear.^[10,42,43]^ In our study, women with GDM stated that “I had fears that my baby would be harmed while pregnant” (P1, 35), “I was worried that my child would be harmed or that I wouldn’t be able to feed my baby enough because my sugar was high or low” (P5, 32), a child will have a problem in their organs’ (P5, 32), “I was worried that harm would occur” (P8, 31). According to Lapolla et al in their research entitled “Quality of life, desires and needs in women with GDM: The Italian DAWN pregnancy study,” they found that immigrant and Italian women with GDM have anxiety and experience fear as the most dominant emotion. Women are especially afraid of the possible consequences for their children. It was concluded that although women were also worried about their general health, they were partially or moderately optimistic during pregnancy.^[36]^ A study conducted by Hui et al was similar to our study. It was reported that women with GDM had anxiety about the health of their babies.^[42]^ In studies conducted by Devsam et al and Miazgowski et al, it was also stated that women with GDM experienced severe fear about the health of their babies.^[10,43]^

Women with GDM blamed themselves for the diagnosis. In previous studies, it is stated that women with GDM mostly blame themselves for the diagnosis and worry about the health of their babies as stressors.^[35,45,46]^ According to Hirst et al in a qualitative study conducted in Vietnam in 2012 to determine the attitudes and health behaviors of women with GDM, women stated that they were diagnosed and blamed because they did not control their diets.^[35]^ Some believed it to be caused by heredity, while others believed it to be caused by stress and medications.^[47]^ In our study, similar expressions were used, such as “I think it was because of my weight and what I ate” (P4, 29), and “I was afraid that I was carrying a problematic gene” (P9, 27). In the same study, pregnant women stated that they feared the risk of preterm birth the most because of their anxiety. Similarly, in our study, the expression “...I was afraid of premature birth...” (P4, 29) was used.

In our study, women with GDM both blamed themselves for the diagnosis and worried about the negative consequences of gestational diabetes for their babies. Premature birth or having a baby with diabetes was just one of them. In addition, women with GDM blamed themselves for not complying with their diets, being malnourished, or being genetic carriers. This result may show that women have a lack of awareness about gestational diabetes, and pregnant women may have negative beliefs and perceptions about diabetes diagnosis. Therefore, we think that women with GDM blame themselves for diabetes and their concerns about their babies increase.

In our study, the concern of women with GDM was not only about the health of their babies during pregnancy. We concluded that another important concern of women with GDM was that babies are also diagnosed with diabetes. Another important concern of women with GDM was the fear of being diagnosed with GDM in their next pregnancy. This caused women to refer to themselves as “problematic carriers of genes” and “problematic genes.” In our research, women said “..I am still afraid that I will have a baby with a risk of diabetes..” (P12, 24), “..I check blood sugar to see if I have diabetes..” (P7, 35), “..my child, I am afraid that he will have diabetes too..” (P6, 23), “..I think he will be diagnosed with diabetes..”(P3, 30), “If it is in our genes, this problem may be in my child’s too. I am raising it with fear” (P2, 33). Similarly, in a qualitative study by Nolan et al in which they investigated women’s gestational diabetes experiences, there were widespread expressions of fear that women could adversely affect the health of their babies prenatally, postpartum, and in the long term. Most women reported feeling guilty, especially when their babies had to undergo additional monitoring and testing after birth. Participants also expressed concern about their baby’s risk of developing type 2 diabetes in the future.^[37]^ In another study with similar results, 44% of those with GDM expressed their fear of harming their child or the risk of developing diabetes in the future during the child’s life.^[34]^ In a study conducted by Parsons et al, it was reported that women with GDM were worried about future diabetes, both for themselves and their babies.^[48]^

### 4.1. Limitations

The main limitation of the study is that the sample of the study consisted only of women with GDM in the endocrine clinics of a private hospital the province where the study was conducted (one city).

## 5. Conclusion

This study used a phenomenological approach to examine the fear of diabetes in the babies of women with GDM. This research shed light on the problems that women with GDM experience with themselves and their babies and how they cope with these problems. The women with GDM had tried to accept and cope with the diagnosis. This research shows that the women were worried about both themselves and their babies. This study also reveals the relationship between the triad of “gestational diabetes,” “pregnancy,” and “baby” in women with GDM. It explained the different dimensions of problems experienced by women with GDM in endocrine clinics. Illuminating the experiences of women with GDM is part of an integrative care approach that will help increase quality care and treatment (service) in endocrine clinics. Thus, the difficulties and problems experienced by women with GDM in endocrine clinics will decrease, and their positive experiences and their satisfaction with health professionals will increase. More qualitative studies are needed to learn more about the experiences of women with GDM in endocrine clinics.

## Acknowledgments

We thank the women who participated in the study.

## Authors contributions

**Conceptualization:** Ekin Dila Topaloğlu Ören, Elif Ünsal Avdal, Gökşen Polat, Funda Sofulu, Gönül Düzgün, Gülseren Pamuk.

**Data curation:** Ekin Dila Topaloğlu Ören.

**Formal analysis:** Ekin Dila Topaloğlu Ören, Elif Ünsal Avdal, Gökşen Polat, Funda Sofulu, Gönül Düzgün, Gülseren Pamuk.

**Supervision:** Ekin Dila Topaloğlu Ören, Elif Ünsal Avdal, Gökşen Polat, Funda Sofulu.

**Writing – original draft:** Ekin Dila Topaloğlu Ören.

**Writing – review & editing:** Ekin Dila Topaloğlu Ören, Elif Ünsal Avdal, Gökşen Polat, Funda Sofulu, Gönül Düzgün, Gülseren Pamuk.
